# Graves’ disease with anti-GQ1b antibody syndrome: a rare case report

**DOI:** 10.1186/s12883-021-02245-1

**Published:** 2021-05-28

**Authors:** Tao Liang, Zhiwei Zhou, Xiaolin Hu, Zhong Luo

**Affiliations:** 1 Department of Neurology, the Affiliated Hospital of Zunyi Medical University, Zunyi, Guizhou China; 2 Department of Surgery Spine, the Affiliated Hospital of Zunyi Medical University, Zunyi, Guizhou China

**Keywords:** Graves’ disease, GQ1b antibody, Limb weakness, Extraocular paralysis, case report

## Abstract

**Background:**

Graves’ disease and anti-GQ1b antibody syndrome are both autoimmune diseases, and there have been few reports on whether there is a correlation between the two. In this study, we present the case of a woman who was diagnosed with Graves’ disease and anti-GQ1b antibody syndrome in succession.

**Case presentation:**

The chief complaints of this patient were limb weakness and blurred vision. Graves’ disease was diagnosed by examination of thyroid function and thyroid autoantibodies, but the clinical symptoms were not relieved after antihyperthyroidism treatment. Finally, it was found that Graves’ disease was complicated by anti-GQ1b antibody syndrome, and the symptoms were relieved after treatment with glucocorticoids and intravenous immunoglobulin. We also explored the possible mechanism of these diseases through a literature review.

**Conclusions:**

We report a rare case of the cooccurrence of Graves’ disease and anti-GQ1b antibody syndrome. Immune dysregulation might be the pathogenesis of the association, but there is no precise supporting evidence, and more research is needed.

## Background

Thyrotoxicosis is a clinical state that results from excessively high thyroid hormone action in tissues, generally due to inappropriately high tissue thyroid hormone levels caused by a variety of etiologies; Graves’ disease (GD) is one of the most common diseases causing thyrotoxicosis [1, 2]. The occurrence of GD is highly correlated with autoimmunity, which, due to abnormal production of thyrotropin receptor antibody by multiple factors, results in high thyroid hormone action in tissues. Anti-GQ1b antibody syndrome was first described by Odaka in 2001 upon summarizing the clinical features of patients positive for serum anti-GQ1b antibody, and this syndrome is a type of autoimmune disease [3]. In terms of pathogenesis, anti-GQ1b antibody syndrome and GD are two different diseases. Although they are both autoimmune diseases, there have been few reports on whether there is a correlation between the two diseases. Here, we report a rare case of GD with anti-GQ1b antibody syndrome encountered at the affiliated hospital of Zunyi Medical University.

## Case presentation

**Fig. 1:**
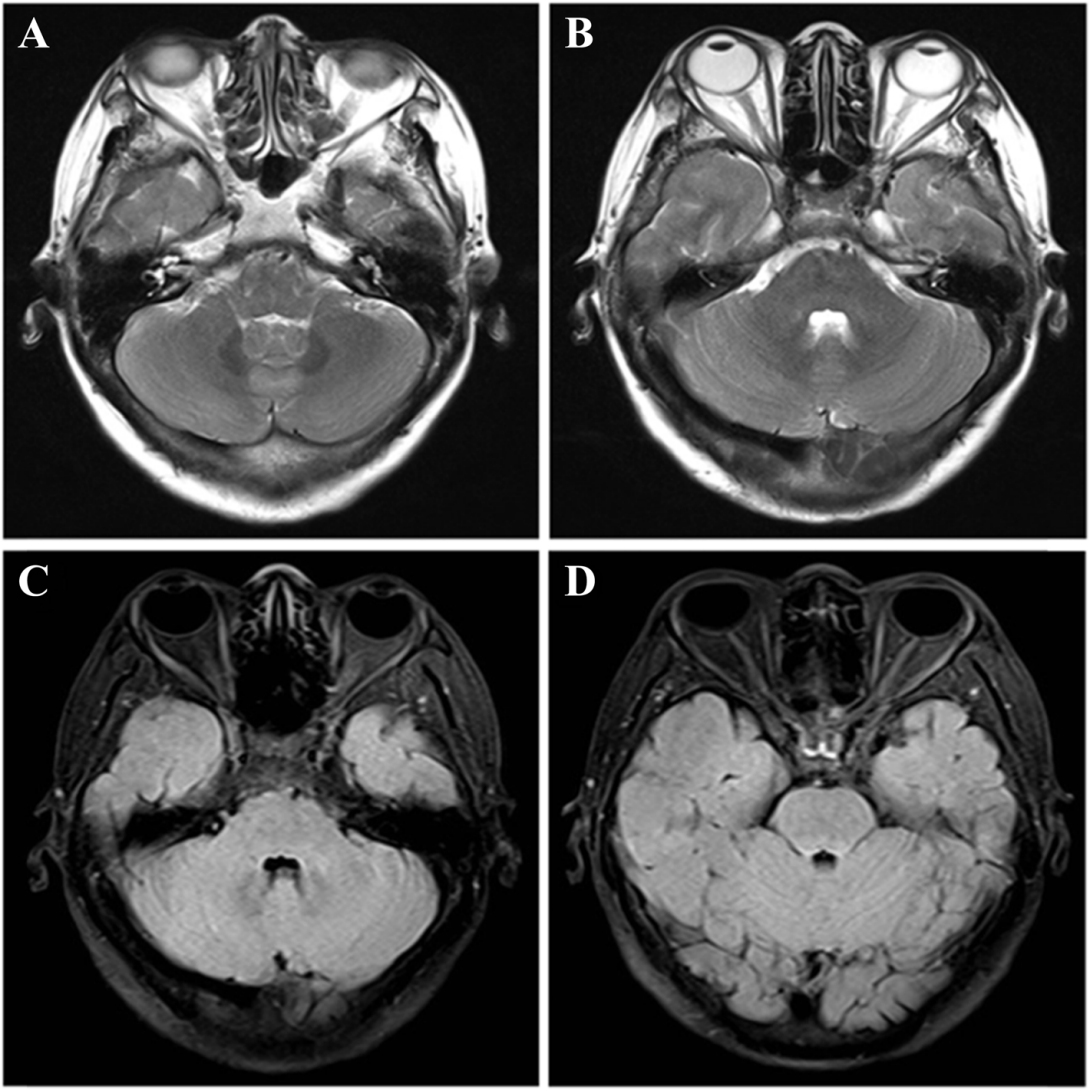
the patient’s MRI of the brain showed no damage of extraocular muscle. (A, B) T2WI axial image. (C, D) T1WI axial image

A 45-year-old female patient was admitted to the endocrinology department of our hospital for blurred vision and weakness in both lower limbs. Three days before admission, the patient had no obvious cause of blurred vision, double shadow, or decreased vision. Two days before admission, the patient had weakness in both lower limbs when she rose in the morning and could walk alone slowly at first. The patient had no history of cold or diarrhea in recent days. There was no numbness, dyspnea, or urinary or rectal dysfunction during the disease period. She had no medical history or family history of any diseases and no history of drug abuse. The patient sought medical advice at the local hospital, and no obvious abnormalities were found on cranial computed tomography (CT) examination. However, the symptoms described above were progressively aggravated, and soon she could not walk independently. She had difficulty lifting her arms and holding objects because of increasing weakness in both upper limbs. Physical examination indicated that bilateral eyeball protrusion, slightly limited bilateral eye movement, and I° thyroid enlargement. The upper extremity muscle force was grade IV, the lower extremity muscle force was grade II, tendon reflexes at the knee and ankle were absent, and there was no extremity sensory disturbance. The laboratory test results were as follows: thyroid-stimulating hormone (TSH): 0.007 μIU/ml (reference range: 0.5–4.8); triiodothyronine (FT3): 29.3 pmol/l (reference range: 2.77–6.31); free thyroxine (FT4): 89.4 pmol/L (reference range: 10.45–24.38); thyroid peroxidase antibody (TPOAb): > 600.0 IU/ml (reference range: 0–34); thyroglobulin antibody (TGAb): > 4000.0 IU/ml (reference range: 0–115); and TSH receptor antibody (TRAb): 5.91 IU/L (reference range: < 1.75). Color Doppler ultrasound showed diffuse enlargement of the thyroid. Given these findings, GD was diagnosed by an endocrinologist, and methimazole and propranolol were administered as an initial treatment for 5 days. Repeat thyroid function tests revealed 9.3 pmol/L FT3 and 42.5 pmol/L FT4, indicating a significant decrease compared with the results of the first test. However, the patient’s limb weakness and blurred vision were not significantly relieved by the changes in thyroxine. The endocrinologist thought that the patient’s clinical features could not be fully explained by GD. The patient was transferred to the neurology department after full discussion with an endocrinologist and neurologist. Lumbar puncture showed that the cerebrospinal fluid (CSF) white blood cell count was determined as 8 × 10^6^/L and the protein level was 886 mg/L. The magnetic resonance imaging (MRI) findings of the cranial region and whole spinal cord were unremarkable, and there was no extraocular muscle damage (Fig. 1). Nerve conduction velocity (NCV) examination showed that the sensory conduction velocity (SCV) of the left median nerve was slowed, the distal motor conduction velocity (MCV) latency was prolonged, the MCV of the elbow segment of the left ulnar nerve was slowed (Table 1), the amplitude of the F wave of the right ulnar nerve and the left tibial nerve was decreased, and the speed of the F wave of the right tibial nerve and the left peroneal nerve was decreased (by 10 and 20%, respectively), indicating demyelination damage. Electromyography showed no denervated potentials or myogenic damage. These results supported the diagnosis of Guillain-Barré syndrome (GBS); impulse therapy with methylprednisolone was given after the exclusion of contraindications, and antihyperthyroidism therapy, vitamin B and rehabilitation were administered as complementary treatments. However, the patient’s muscle strength and blurred vision were not obviously relieved. Lumbar puncture was performed again, and CSF and peripheral blood were collected for the detection of peripheral nerve disease-related antibodies. The results were positive for CSF GQ1b antibody and serum GQ1b, GT1a, and GM1 antibodies. With full consideration of the clinical course and features, as well as the auxiliary examination results, anti-GQ1b antibody syndrome was diagnosed, and intravenous immunoglobulin was administered continuously for 5 days. Fortunately, the patient’s blurred vision and limb muscle strength gradually improved, and she recovered unlimited eye movement in all directions. She was transferred to the rehabilitation department for further treatment; grade 5 and 3 muscle strength was present in both upper limbs and lower limbs, respectively. At the 3-month follow-up, her muscle strength was grade 4 in both lower limbs.

**Table 1:** The patient’s NCV test findings

Nerve/Site	SCV				MCV				
	Onset Latency (ms)	NP Amplitude (μV)	Distance (mm)	Velocity (m/s)	Electrode	Latency	Amplitude (mV)	Distance (mm)	Velocity (m/s)
Median Nerve(L)	3.02	5.1	145	**48**	Wrist	**4.48**	5.6		
					Elbow	7.29	5.2	175	62
					Axilla	8.91	5.1	120	72
Median Nerve(R)	2.92	5.0	146	50	Wrist	3.65	7.4		
					Elbow	6.82	7.6	182	57
					Axilla	8.91	7.2	125	60
Ulnar Nerve(L)	2.45	4.7	129	53	Wrist	2.34	4.0		
					Elbow	6.93	3.2	85	**37**
					Axilla	8.91	3.8	115	110
Ulnar Nerve(R)	2.34	4.4	133	57					

## Discussion and conclusion

In this case, the diagnosis of GD was clear based on the clinical manifestations and auxiliary examination results, but the limb weakness failed to improve with the use of antihyperthyroidism drugs. Examination of the CSF was showed protein-cell separation phenomena, and NCV examination indicated demyelination damage to peripheral nerves. Additionally, both the CSF and serum were positive for GQ1b antibody. According to the literature, anti-GQ1b syndrome is known as a spectrum disorder with a common autoantibody (anti-GQ1b IgG), yet its clinical symptoms are not specific. Forming a continuous range, it includes Miller-Fisher syndrome, acute ophthalmoparesis, GBS, and GBS with extraocular paralysis [3]. It has also been reported that decreased vision, ophthalmoplegia and peripheral facial paralysis are the main manifestations [4]. Although there was more than one type of antibody in the patient’s serum, this situation is not rare according to related reports of anti-GQ1b antibody syndrome [5–7]. Due to their close structural relationship, anti-GT1a antibodies often cross-react with anti-GQ1b antibodies. This cross-reactivity usually results in similar anti-GQ1b and anti-GT1a titers and is correlated with ophthalmoparesis [7]. Anti-GM1 antibodies widely express ganglioside and are most frequently associated with acute motor axonal neuropathy (AMAN) and multifocal motor neuropathy (MMN), serving as a pathophysiological link between these acute and chronic immune neuropathies [8, 9]. A reasonable guess is that the titer of anti-GQ1b antibody may have been higher than that of other antibodies because of the positive result in both the serum and CSF. Therefore, anti-GQ1b antibody syndrome was most likely the cause of the symptoms, and combined with the clinical manifestations and other findings, this case was classified as GBS with extraocular paralysis. Eye movement disorder or limb weakness could be the result of GD and anti-GQ1b antibody syndrome, but in terms of pathogenesis, GD and anti-GQ1b antibody syndrome are two completely different diseases, and the relationship between them is not clear. In this case, the patient’s eye movement abnormalities and limb weakness may have been induced by anti-GQ1b syndrome because no orbital edema related to hyperthyroidism was revealed on brain MRI and because the patient’s chief complaint was relieved by immunotherapy rather than antihyperthyroidism therapy. To date, the prevalence of GQ1b antibody, a member of the ganglioside antibody family, in humans is unknown. Gangliosides are glycosphingolipids characterized functionally and structurally by polymorphic sialic acids and are widely distributed in the human body. They play an important role in protecting humans from immune attacks; equally, they can become targets for autoimmunity, leading to autoimmune diseases [10]. Currently, it is thought that molecular simulation is the main pathogenic mechanism in anti-GQ1b syndrome, which means that GQ1b antibody induced by different causes attacks specific GQ1b sites in the peripheral and central nervous systems. A literature review showed that GQ1b antibody acts as a receptor for *Campylobacter jejuni*, *Haemophilus influenzae* and other bacteria, as well as viruses; that is, these microbes have an antigenic structure similar to that of GQ1b to the human body [11–13]. GD is an autoimmune disease caused by unhealthy TRAb levels and characterized by excess thyroid hormone synthesis and secretion by the thyroid gland [14]. Excess thyroid hormone affects almost all organ systems of the body and is not limited to the thyroid. Commonly reported neuromuscular symptoms are tremor, nervousness, anxiety, fatigue, weakness, disturbed sleep, and poor concentration. Excess thyroid hormone can also lead to hyperreflexia and myasthenia, which are mainly seen in cases of hypokalemic periodic paralysis and pelvic and girdle muscle weakness, but not hyporeflexia, and muscle force usually recovers in a short time after antihyperthyroidism treatment. GD can be associated with other systemic autoimmune diseases in the same patient. Silvia reviewed 3209 GD patients and found that approximately 16.7% had another associated autoimmune disease, but without any cases of anti-GQ1b antibody syndrome [15]. There have been some case reports of GD combined with GBS, GD with a rare pharyngeal-cervical-brachial variant of GBS, GD with the development of postinfectious GBS during a course of methimazole-induced agranulocytosis, and GD manifesting as periorbital swelling, double vision, hyperosmia, and dizziness; in this last case, the level of anti-GQ1b antibody was elevated (> 15,000 U), suggesting Miller-Fisher syndrome [16–18]. However, our subject was newly diagnosed, had different clinical symptoms, and was not treated with any antithyroid drug at the time of presentation. An animal study has shown that pathophysiological changes in the thyroid gland affect the biosynthesis and expression of ganglioside in specific tissues and cell types, which can lead to abnormal ganglioside levels in rats [19]. Using specific monoclonal A2B antibody immunostaining, Saito found ganglioside antibody expressed in the thyroid, pancreas, kidney and other extraneural areas [20].

Generally, there have been few studies and clinical reports on the relationship between GD and anti-GQ1b antibody syndrome. It is not clear whether the relationship between the two was causal, and while autoimmunity is the leading cause of the development of both of these diseases, the exact mechanism of this association is not well understood. Thus, more basic studies and clinical reports are needed to confirm the relationship between them.

## Data Availability

The datasets used or analyzed during the current study are available from the corresponding author on reasonable request.

## Abbreviations

GD: Graves’ disease
CT: Computed tomography
MRI: Magnetic resonance imaging
TSH: Thyroid stimulating hormone
FT3: Free T3
FT4: Free T4
TPOAb: Thyroid peroxidase antibody
TGAb: Thyroglobulin antibody
TRAb: Thyrotropin receptor antibody
HIV: Human immunodeficiency virus
TPPA: Treponema pallidum particle agglutination assay
SCV: Sensory nerve conduction velocity
MCV: Motor nerve conduction velocity
CSF: Cerebrospinal fluid
AMAN: Acute motor axonal neuropathy
MMN: Multifocal motor neuropathy
